# Low Bacterial Diversity and High Labile Organic Matter Concentrations in the Sediments of the Medee Deep-Sea Hypersaline Anoxic Basin

**DOI:** 10.1264/jsme2.ME12045

**Published:** 2012-04-14

**Authors:** Ioanna Akoumianaki, Hidetaka Nomaki, Maria Pachiadaki, Konstantinos Ar. Kormas, Hiroshi Kitazato, Hidekazu Tokuyama

**Affiliations:** 1 Institute of Oceanography, Hellenic Centre for Marine Research, Anavissos 19013, Attiki, Greece; 2 Institute of Biogeosciences, Japan Agency for Marine-Earth Science and Technology (JAMSTEC), 2–15 Natsushima-cho, Yokosuka 237–0061, Japan; 3 Department of Ichthyology and Aquatic Environment, School of Agricultural Sciences, University of Thessaly, 384 46 Nea Ionia, Greece; 4 Atmosphere and Ocean Research Institute (AORI), University of Tokyo, 5–1-Kashiwanoha, Kashiwa, Chiba 277–8564, Japan

**Keywords:** DHAB, hydrolyzable biopolymers, organic carbon, 16S rRNA, sediment

## Abstract

Studies in the center and margin of the Medee Basin, a Mediterranean deep-sea hypersaline anoxic basin, and at a reference site during Penelope cruise (2007), revealed the existence of a 7 m-thick halocline, with high salinity (328 psu), and high sedimentary organic carbon and biopolymer concentrations. The 194 16S rRNA sequences retrieved were grouped into 118 unique phylotypes. *Pseudomonas gessardii*, dominated in the center, while 33 phylotypes were detected at the margin and 73 at the reference site. The study suggested conditions hostile to bacteria in the sediments of the Medee Basin and preservation of sedimentary labile organic matter.

The deep-sea hypersaline anoxic basins (DHABs) identified thus far across the Mediterranean Ridge probably resulted from the dissolution of buried Messinian evaporitic deposits that became exposed to seawater after tectonic shifts and were then entrapped into local abyssal depressions (4, 10, 12, 30, 31). Salinity in these DHABs is up to 5–10 times that of normal seawater with the ions Na^+^, K^+^, Ca^2+^, Mg^2+^, Cl^−^ and SO_4_^2−^ and methane comprising the main solutes (6, 31). Precipitation of calcium carbonate, biogenic opal and gypsum has also been reported (5, 30). In each of these DHABs the prokaryotic community structure and function in the brine-seawater interface were found to be directly linked to brine geochemistry (7, 15, 28).

The Medee Basin (Fig. S1A) was discovered in 1995 (4). Although it is the biggest DHAB ever found on Earth (4), it was not investigated until 2007. The present study explored the quantity and composition of organic matter (OM) and the bacterial 16S rRNA gene diversity in sediments at the center and margin of the Medee Basin with an eye toward stratification of the overlying water column, for which no previously published data are available.

Sampling took place from 26 January to 5 February 2007 onboard the R/V Hakuho Maru. Mapping with air-gun seismic profiles revealed an elongate depression (length ~50 km, area ~112 km^2^) comprising five sub-basins of different dimensions and depths, ranging from 3,058 m in the western to a maximum depth of 3,101 m in the eastern sub-basin (Fig. S1B).

A Navigable Sampling System (NSS), an ROV system equipped with two TV cameras, Niskin bottles, piston corer, altimeter and thrusters developed by AORI, was used prior to sampling to detect micro-environments within 2 m accuracy. Three sites were selected to study the sedimentary environment of the Medee DHAB (Fig. S1C): the inside margin (IMS) of the DHAB at 2,938 m depth, the central part (CS) of the western sub-basin of the DHAB at 3,060 m depth, and the outside margin (OMS) of the DHAB at 2,910 m depth. A nearby reference location (RS) at 2,750 m depth, not influenced by anoxic brine or mud volcanoes, was also sampled (Fig. S1C).

Salinity (PSU) and temperature (°C) were obtained with CTD (Sea Bird, SBE9plus) casts at all sites, while dissolved oxygen (DO) concentration was determined only at CS and OMS. Salinity of the brine was additionally measured with a salinometer by diluting bottom water sampled with the CTD water sampling system. Undisturbed sediment samples were taken using a mini-multicorer equipped with GPS (ORI) at each site. Sediment core samples were sub-divided on board into three sediment depth layers: (0–5) cm, (5–10) cm and (10–20) cm. About 50–100 mL of wet homogenized sediment slurries were sampled from independently collected sediment cores. These were then stored in pre-combusted aluminium foil at −80°C for the integrated study of total organic carbon (TOC) and biopolymeric compounds, *i.e.*, proteins and carbohydrates, upon return to the laboratory. Top 2 cm sediment samples were kept at 4°C prior to microscopic observations. Sediment samples for prokaryotic community analyses were collected from the (0–5) cm layer under sterile conditions and were directly sealed and stored at −80°C.

Sediment biochemical parameters were measured using three freeze-dried homogenized aliquots. TOC was estimated as in Nelson and Sommers (21). Total protein (PRT) was determined according to Hartree (16) modified by Rice (25) to compensate for phenol interference, following extraction with NaOH (0.5 M, 4 h). Concentrations were expressed as bovine serum albumin equivalents. Total carbohydrate (CHO) was analyzed according to Gerchacov and Hatcher (13) and expressed as glucose equivalents. Enzymatically hydrolyzable proteins (HPRT) and carbohydrates (HCHO) were determined in aliquots from sediment slurries according to Dell’Anno *et al.* (11) without modification. For HPRT, aliquots were incubated in Na-phosphate buffer (0.1 M; pH 7.5) with proteinase K (1 mg mL^−1^) and protease (600 μg mL^−1^) for 1 h at 37°C. For HCHO, aliqots were incubated in 0.1 M Na-phosphate, 0.1 M EDTA buffer (pH 6.9) with α-amylase, β-glucosidase, proteinase-K and lipase (from stock solutions 1 mg mL^−1^ for each enzyme) for 1 h at 25°C. Blanks were not incubated with enzymes, and were muffled at 550°C for 4 h. Differences between blank and enzymatically treated subsamples were assumed to represent the concentrations of HPRT and HCHO. All concentrations were normalized to sediment dry weight. Differences in TOC, HPRT and HCHO concentrations among sites and sediment layers at each site were tested with one-way ANOVA, followed by SNK multiple comparisons.

DNA from RS, CS and OMS was extracted from 0.5–1 g sediment using the UltraClean Soil DNA kit (MoBio Laboratories, Carlsbad, CA, USA) with minor modifications to the manufacturer’s protocol. Specifically, bead beating was reduced from 10 to 5 min and was immediately followed by three cycles of freeze-and-thaw (−80°C for 3 min and then immediately in 65°C water bath for 5 min) after addition of the inhibitor removal solution. Bacterial 16S rRNA gene was amplified using the bacterial primers 27f (5′-AGA GTTTGATCCTGGCTCAG-3′) and 1390r (5′-GACGGGCG GTGTGTACA-3′). PCR included an initial denaturation step at 94°C for 1 min followed by 25 to 29 cycles consisting of denaturation at 94°C for 45 s, annealing at 52.5°C for 45 s, and elongation at 72°C for 2 min; a final 7-min elongation step at 72°C was added. The number of cycles was determined for each sample after cycle optimization. PCRs were repeated with different cycle numbers, and the lowest number of cycles that gave a positive signal was then used for cloning and sequencing in order to avoid differential representation of 16S rRNA gene with low and high copy numbers. Six tubes of PCR products were pooled to reduce the biases of each individual reaction.

PCR products were visualized and stained with EtBr on a 1% agarose gel under UV light to excise bands. DNA was extracted with the PureLink Quick Gel Extraction Kit (Invitrogen Corporation, Carlsbad, CA, USA) following the manufacturer’s protocol. PCR products were cloned using the TOPO TA for sequencing cloning kit (Invitrogen) using electrocompetent cells according to the manufacturer’s specifications. For each sample, randomly picked clones with inserts of the expected length were analyzed. Clones were grown in liquid LB medium with kanamycin and their plasmids were purified using the NucleoSpin Plasmid QuickPure kit (Macherey-Nagel, Düren, Germany) for DNA sequencing.

Sequence data were obtained by Macrogen (Seoul, South Korea) using capillary electrophoresis and the BigDye Terminator kit (Applied Biosystems Carlsbad, CA, USA) with the primers M13F(-20) and M13R. Every sequence read was approximately 900 bp and for each individual clone, forward and reverse reads were assembled. The sequences were screened for chimeras using Pintail (http://www.bioinformatics-toolkit.org/Web-Pintail/) and Bellerophon software (http://greengenes.lbl.gov/cgi-bin/nph-bel3_interface.cgi). All putative chimeras were excluded from further analysis.

All sequences were compared with the BLAST function (http://www.ncbi.nlm.nih.gov/BLAST/) for their closest relative. Sequence alignment was performed using the SILVA alignment utility (http://www.arb-silva.de/aligner/). Phylotypes were defined as sequences showing ≥98% homology to each other, using CLUSTALW (http://www.ebi.ac.uk/Tools/msa/clustalw2/). Phylogenetic trees were constructed by the neighbor-joining method using the Jukes-Cantor correction. Neighbor-joining bootstrap analyses for 1,000 replicates were performed to assign confidence levels to the tree topology using MEGA4 software (26). Clone library coverage was calculated by the Good’s C estimator, a nonparametric estimator of the proportion of phylotypes in a library of infinite size that would be represented in a smaller library (14, 19).

A 7 m-thick halocline, in which salinity increased from 42.01 to over 99 PSU (upper limit of salinity sensor), at 2,917 m depth separated a 160 m-deep anoxic brine from the overlying seawater (Fig. S2A and B). Across this sharp halocline, temperature increased by 0.44°C, whereas DO dropped to anoxic levels. Brine salinity reached ca. 328 psu immediately above the sea bottom. A 49 m-thick layer, through which salinity rose by 1.9 psu, but DO declined from 4.16 to 2.7 mL L^−1^, lay above the halocline (Fig. S2A and B). On the other hand, maximum salinity at the OMS site did not exceed 39.06 psu, indicating that, at least during our sampling, the hypersaline brine did not reach the OMS depth (Fig. S2B). That said, the water column above the OMS depth was homogeneous, in terms of temperature and salinity, in the whole study area (data not shown).

IMS and CS sediments were dark grey in contrast to dark olive at OMS and olive yellow at RS. A layer of gypsum crystals was found at CS (20 cm sediment depth). Coccoliths and tests of foraminifera and silicoflagellates were found at all sites.

TOC increased with sediment depth (*P*<0.05) at all sites and reached 2.03% at OMS, 2.56% at IMS and 3.4% at CS in contrast with only 0.5% at RS at the 10–20 cm sediment layer (Fig. 1A). TOC concentrations significantly increased from the RS to the CS site in all sediment layers (*P*<0.05).

Total PRT and CBO were higher at IMS and CS than at RS (Fig. 1B and C, respectively). In addition, all sediment layers displayed significantly higher HPRT at CS and IMS than at OMS and RS (F_(0−5)_=284, F_(5−10)_=85, F_(10−20)_=51, *P*<0.05). HPRT increased with sediment depth at OMS, IMS and CS (F_OMS_=2, F_IMS_=17, F_CS_=34, *P*<0.05), but decreased with sediment depth at RS (F_RS_=19, *P*<0.05). HCBO increased with sediment depth at the OMS, IMS and CS sites (F_OMS_=90, F_IMS_=41, F_CS_=11, *P*<0.05), while no such changes could be detected at RS. Morevoer, HCBO increased from the RS site to the OMS, IMS and CS at all sediment layers (F_(0−5)_=75, F_(5−10)_=189, F_(10−20)_=135, *P*<0.05).

Overall, 194 sequences were analyzed and grouped into 118 unique phylotypes. Clone library coverage was low for RS and OMS but high for the CS sample (Fig. S3). Only two phylotypes were found at CS, both affiliated with *Gammaproteobacteria*, (Fig. S4). CS-B1 dominated (30/31 clones in the sample) and was closely related to *Pseudomonas gessardii*. Thirty-three unique phylotypes were found at OMS (Fig. S5) with most belonging to the *Alpha*- (α-), *Beta*- (β-), *Gamma*- (γ-), *Delta*- (δ-) and *Epsilon*- (ɛ-)*proteobacteria*. The most abundant phylotypes (OMS-B1 and OMS-B40, 31/73 and 6/73, respectively) belonged to the *Epsilonproteobacteria* and were related with phylotypes from coastal sediments. Four *Gammaproteobacteria* phylotypes were identical to *Legionella pneumophila*, *Pseudomonas gessardii*, *Shewanella putrefaciens* and *Rheinheimera* sp. The rest of the phylotypes were not related to known species and were similar to phylotypes from various shallow and deep-sea sediments and terrestrial habitats (soil, groundwater, waste-water treatment plant).

The 73 retrieved unique phylotypes in RS were grouped into 16 taxa at the phylum level, with nine being novel (Fig. S6). A total of 28.9% of these belonged to the *Alpha*-, *Gamma*- and *Deltaproteobacteria* (Fig. S7) and were not related to any known cultivated taxa but all the *Deltaproteobacteria* phylotypes belonged to the *Myxococcales*. The most abundant non-*Proteobacteria* phylotypes belonged to *Chloroflexi* (19.7%) and *Actinobacteria* (15.8%) and the rest of the phylotypes belonged to the *Acidobacteria*, *Nitrospirae*, *Bacteroidetes*, *Planctomycetes*.

Regarding common phylotypes (Fig. 2), only CS-B1 was found at all sites. Another five phylotypes co-occurred between RS and OMS. Three (RS-3, -27, -77) belonged to novel clades, and two (RS-11, -66) fell within the *Proteobacteria*.

The higher TOC and biopolymer concentrations at the Medee, compared to RS, indicated the transport of fresh OM into the basin and subsequent preservation in the anoxic sediments. This could not be explained by the low OM flux, which is dominated by coccolithophorids in the vicinity of the Medee Basin, *i.e.*, South Ionian Sea (33). However, trenches and deep-sea basins may function as traps of fresh OM when conditions, such as vertical mixing of the water column and enhanced Saharan dust input, allow for simultaneous high phytoplankton production and fast transport to the deep sea (2, 12, 33). In this case, selective sorptive preservation of the hydrolyzable material in fine OM-grain aggregates (18) would further protect OM from breakdown during transport to Medee sediment.

OM composition could also be altered if OM particles were retained across a several meters thick pycnocline long enough to enable efficient degradation (27); however, the Medee seawater-brine halocline was only 7 m thick and about 1 to 4 m thicker than the halocline in other DHABs situated on the Mediterranean Ridge (7). That said, at L’Atalante DHAB, a food web of higher trophic levels, *i.e.*, bacterivorous protists and multicellular eukaryotes, was supported by chemoautotrophic microbes at the oxic-anoxic seawater-brine interface (1, 32). If this is also happening in Medee, then the fallout of the biomass produced across the pycnocline into the brine and its deposition onto the sediments might have contributed to the observed high fraction of hydrolyzable biopolymers.

Once deposited in the anoxic sediments, hydrophobic interactions and hydrogen bonding of peptides in detrital material could provide resistance to degradation and denaturing agents, leading to long-term preservation of proteinaceous aggregates (22). Furthermore, marked colour changes in the layers of a core retrieved from the margin of the brine (Izumitani, N. *et al.* 2010. Abstracts for the Japan Geoscience Union Meeting. APE025-P07 [CD-ROM version, http://wwwsoc.nii.ac.jp/jepsjmo/cd-rom/2010cd-rom/earth2010_disc1/program/PDF/A-PE025/APE025-P07_e.pdf], Chiba) suggested changes in the extent of the brine in the past. This could explain the higher TOC and biopolymer levels in the subsurface layers (5–10 and 10–20 cm) at OMS compared to RS.

Organic enrichment at the sediment surface has also been observed in other DHABs situated on the Mediterranean Ridge, such as the Discovery and Bannock basins (17, 30). Furthermore, protein and carbohydrate concentrations in the Medee were similar to those measured in L’Atalante DHAB sediments (8) and productive coastal areas (*e.g.*, 24), but higher than in other deep-sea sediments (9, RS: present study). Finally, the well-preserved coccoliths and foramniferal tests in the Medee Basin provide evidence of calcite preservation. By contrast, the corrosive brine in the Discovery DHAB sediments resulted in the dissolution of calcareous tests of coccolithofores and foraminifera (30); however, comparisons between Medee and Discovery DHABs regarding siliceous microfossil preservation are impossible because of the relatively shallow core sampling of the present study (*i.e.*, down to 2 cm sediment depth).

The occurrence of phylotypes affiliated with known species could infer similar metabolic features. In this context, the dominance patterns of the phylotypes detected at each site were examined in relation to the species composition and the environmental conditions at each site, in order to make inferences about the ecophysiological role of the bacterial communities at the different sites. Only one phylotype dominated inside the brine and was identical to *P. gessardii*. This is an asporogenous, rod-shaped species with a mesophilic temperature optimum (30°C, range 4–35°C) and significant growth in low-salt concentrations (up to 0.8%) and no growth at 5 or 7% (29). This renders it metabolically inactive inside the brine. In addition, the occurrence of *Pseudomonas*-like cells, along with a few more nonhalophilic *Firmicutes* and *Gammaproteobacteria*, was confirmed in enrichment efforts from the same site (Kormas and Miroshnichenko, unpublished data), supporting the notion that *P. gessardii* could be a contaminant of the sediment sample of unknown origin. This finding, along with the fact that no archaeal DNA amplification was feasible (data not shown), suggest that the Medee sedimentary environment is rather hostile to prokaryotic life. Therefore, OM preservation could be partly attributed to the absence of Bacteria, which otherwise would have at least partially degraded some of the existing organic matter.

By contrast, the bacterial community was richer at the OMS and RS sites. At OMS, it was dominated by the *Epsilonproteobacteria* OMS-B31. Its closest relatives are not extremophilic and it is possibly involved in OM and sulphur cycling, as is the case for many members of this subphylum (3). At RS, the species found belonged either to known phyla or candidate divisions known to dominate in deep-sea sediments (*e.g.*, 20, 23).

In summary, the first investigation of the Medee Basin revealed the hostile features of this extreme habitat for bacterial life. The Medee Basin is one of the most saline DHABs in the Eastern Mediterranean, with total organic carbon concentrations two to eight times higher than in normal deep-sea sediments or other DHABs. Enzymatically hydrolyzable biopolymer concentrations in the Medee sediments were twice as high as at the reference site. These results imply the preservation of OM in the brine, presumably owing to anoxia, hypersalinity and the accumulation of labile material. The evidence of higher microbial diversity at the margin site and of the production of labile material in the halocline should be further investigated to elucidate the marine biodiversity associated with the Medee Basin.

Sequences of unique phylotypes found in this study have GenBank numbers JF809687–JF809794.

## Supplementary Material



## Figures and Tables

**Fig. 1:**
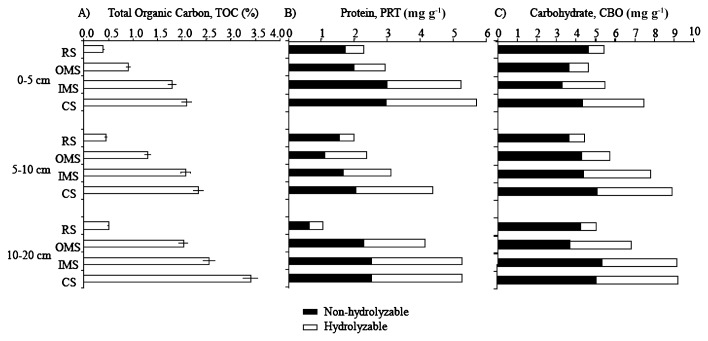
Sediment depth profiles of A) total organic carbon (TOC), B) total and hydrolyzable protein (PRT and HPRT), and C) total and hydrolyzable carbohydrate (CHO and HCHO) at the studied sites.

**Fig. 2:**
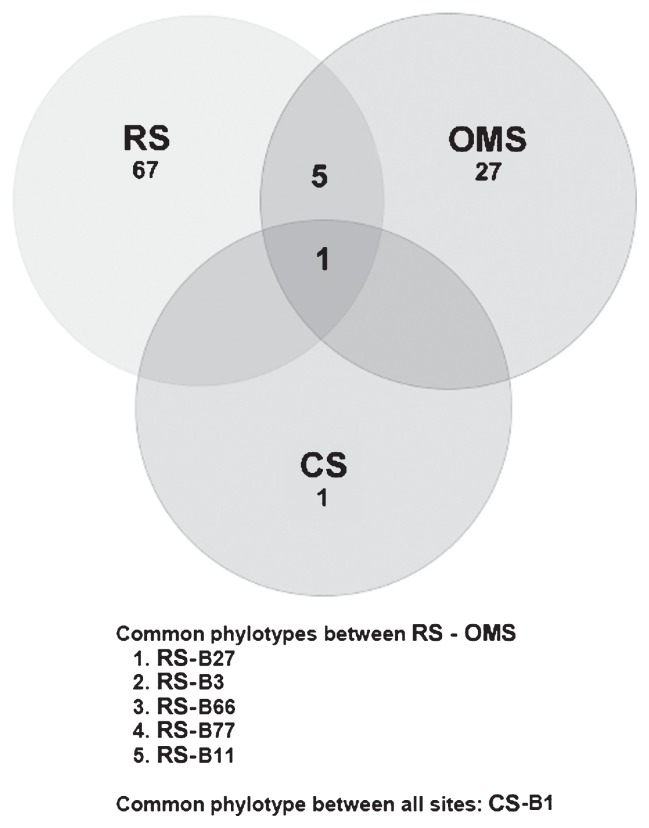
Venn diagrams of the bacterial phylotypes found at the sediment of the three sites of the present study (South Ionian Sea, Medee Basin). The numbers in the circles are the numbers of common phylotypes found between the overlapping circles.
