# Supplementary material for: Low Bacterial Diversity and High Labile Organic Matter Concentrations in the Sediments of the Medee Deep-Sea Hypersaline Anoxic Basin
Source: Microbes Environ. 2012 Apr 14;27(4):504–8. doi: 10.1264/jsme2.ME12045 (PMC4103561; doi:10.1264/jsme2.ME12045)
Supplement: Supplementary file 1 [file 27_504_s1.pdf]

Supplementary materials

Fig. S1. A) Location of the Medee DHAB. B) Medee outline showing the sub-basins. C) Bathymetric map showing locations of sampling sites. RS: Reference Site; OMS: Outside Margin Site; IMS: Inside Margin Site and CS: Central Site in the western sub-basin of the Medee DHAB.

Fig. S2. A) Depth profiles of salinity, temperature and dissolved oxygen (DO) in the water column at the location of CS site. (B) Position of halocline with regard to the depth, shown by arrows, at the Reference Site (RS), Outside Margin Site (OMS), Inside Margin Site (IMS) and the centre of the western sub-basin of Medee (CS).

Fig. S3. Clone library coverage based on Good's C estimator of the bacterial 16S rRNA gene libraries from the Medee DHAB.

Fig. S4. Phylogenetic tree of the Bacteria 16S rRNA gene phylotypes (ca. 1400 bp) in the centre (CS) of the Medee Basin, based on the neighbour-joining method as determined by distance using the Jukes-Cantor two-parameter correction. The found phylotypes (bold letters) are named after the sediment depth origin. Numbers of identical (≥98% sequence similarity) phylotypes of the total phylotype number in each sediment depth are shown in parentheses. One thousand bootstrap analyses (distance) were conducted, and percentages ≥50% are indicated at nodes. Numbers in brackets are GenBank accession numbers. Scale bar represents 2% estimated distance. No number indicates that these phylotypes are singletons.

Fig. S5. Phylogenetic tree of the Bacteria 16S rRNA gene phylotypes (ca. 1400 bp) at the outside margin of the Medee Basin, OMS site in the South Ionian Sea, based on the

neighbour-joining method as determined by distance using the Jukes-Cantor two-parameter correction. The found phylotypes (bold letters) are named after the sediment depth origin. Numbers of identical (≥98% sequence similarity) phylotypes of the total phylotype number in each sediment depth are shown in parentheses. One thousand bootstrap analyses (distance) were conducted, and percentages ≥50% are indicated at nodes. Numbers in brackets are GenBank accession numbers. Scale bar represents 2% estimated distance. No number indicates that these phylotypes are singletons.

Fig. S6. Phylogenetic tree of the Bacteria (excluding *Proteobacteria*) 16S rRNA gene phylotypes (ca. 1400 bp) at the reference site (RS) of the present study based on the neighbour-joining method as determined by distance using the Jukes-Cantor two-parameter correction. The found phylotypes (bold letters) are named after the sediment depth origin. Numbers of identical (≥98% sequence similarity) phylotypes of the total phylotype number in each sediment depth are shown in parentheses. One thousand bootstrap analyses (distance) were conducted, and percentages ≥50% are indicated at nodes. Numbers in brackets are GenBank accession numbers. Scale bar represents 2% estimated distance. No number indicates that these phylotypes are singletons.

Fig. S7. Phylogenetic tree of the *Proteobacteria* 16S rRNA gene phylotypes (ca. 1400 bp) at the reference site (RS) of the present study based on the neighbour-joining method as determined by distance using the Jukes-Cantor two-parameter correction. The found phylotypes (bold letters) are named after the sediment depth origin. Numbers of identical (≥98% sequence similarity) phylotypes of the total phylotype number in each sediment depth are shown in parentheses. One thousand bootstrap analyses (distance) were conducted, and percentages ≥50% are indicated at nodes. Numbers in brackets are GenBank accession

numbers. Scale bar represents 2% estimated distance. No number indicates that these phylotypes are singletons.

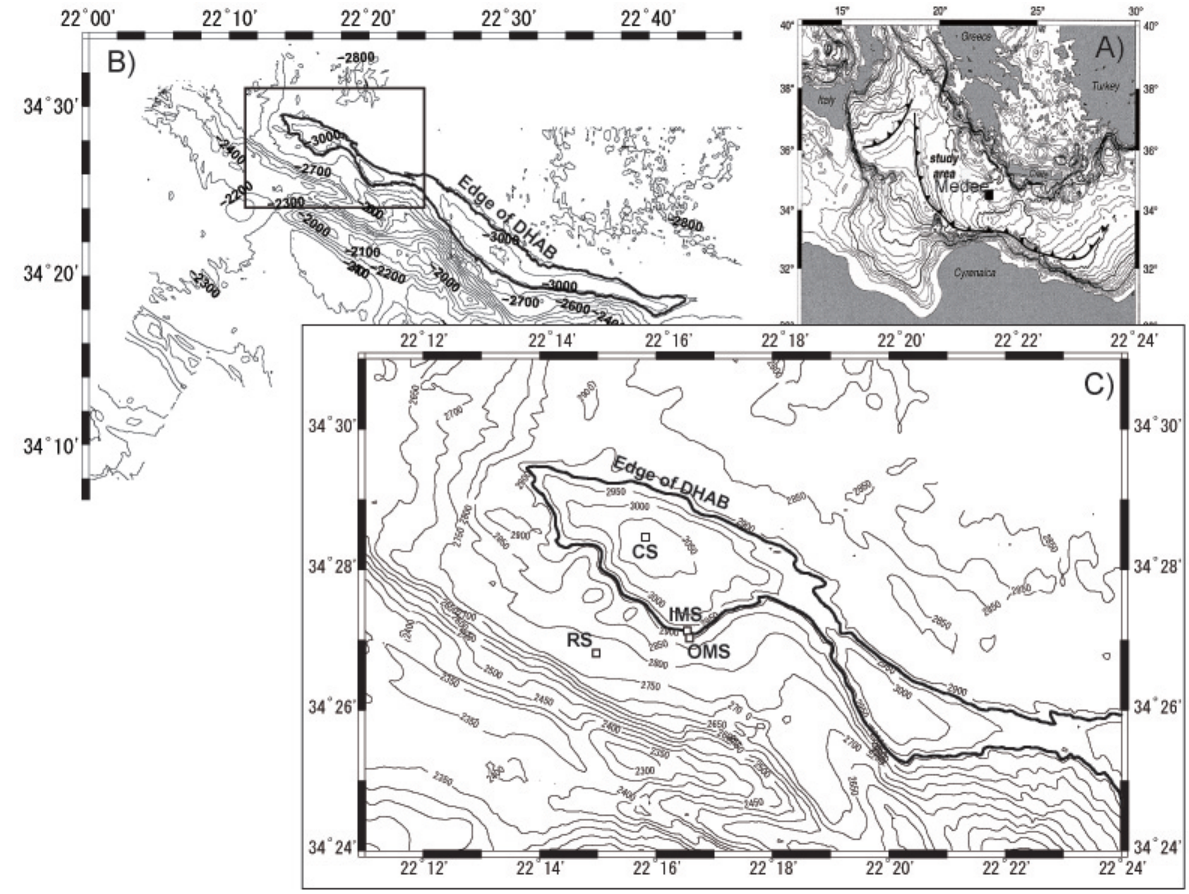

Fig. S1 Akoumianaki

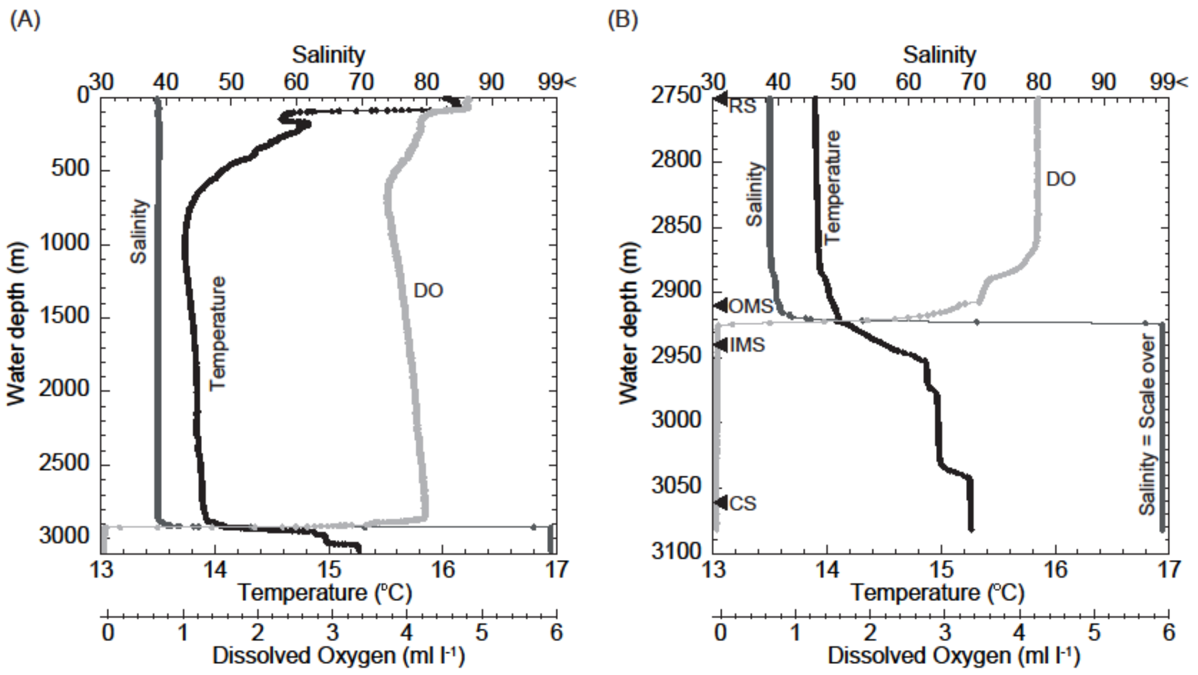

Fig. S2 Akoumianaki

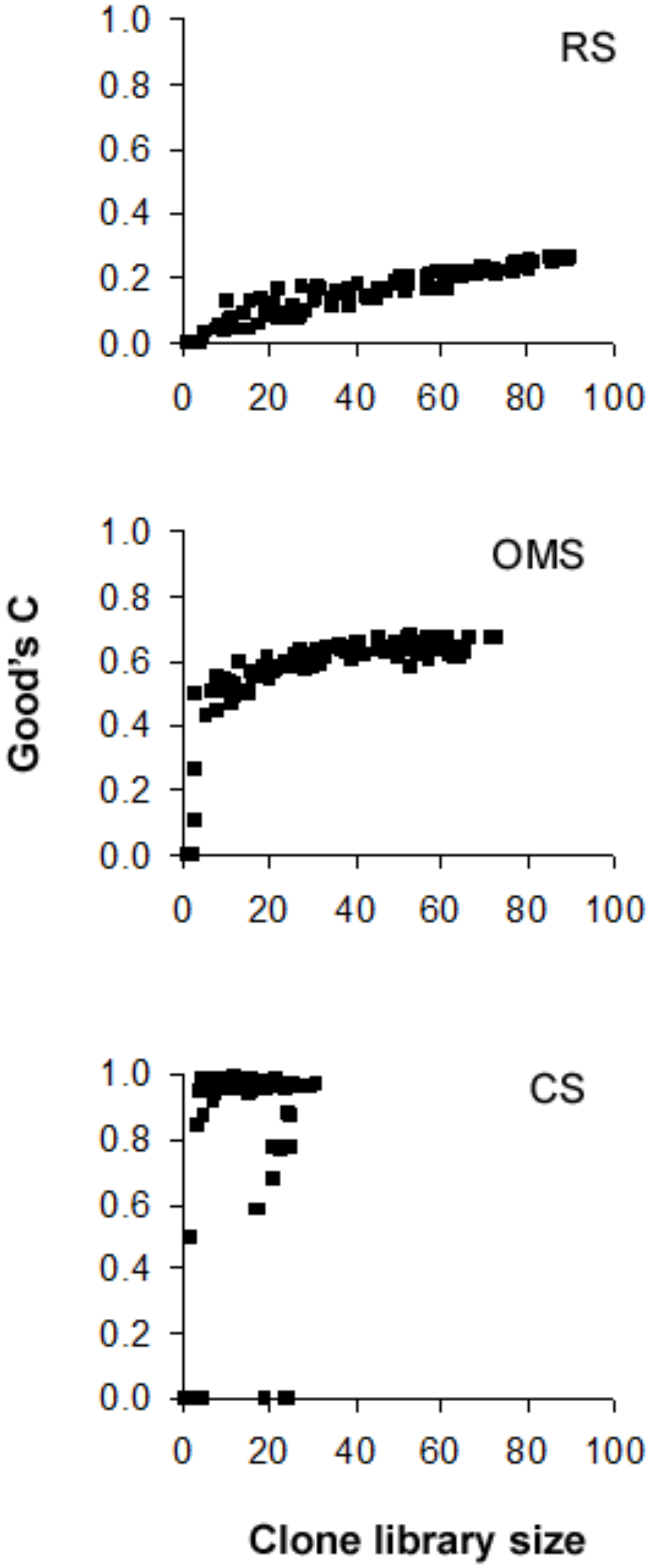

Fig. S3 Akoumianaki

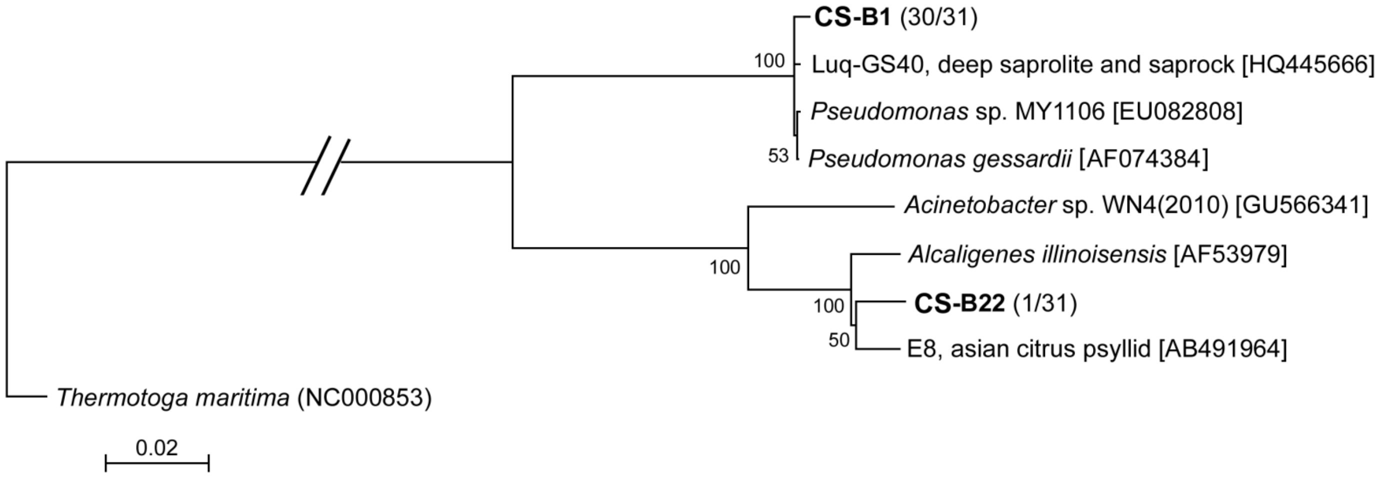

Fig. S4   Akoumianaki

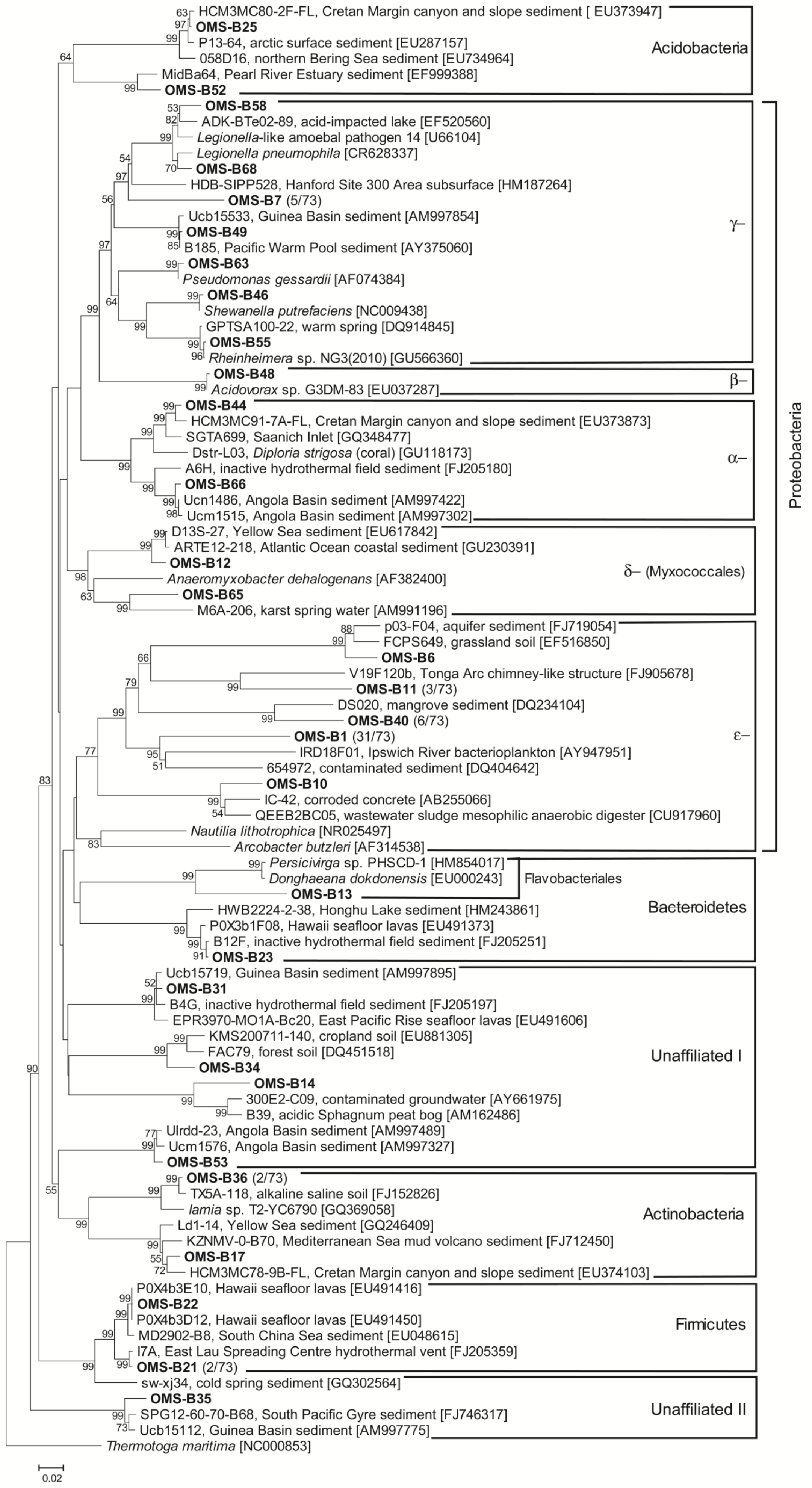

Fig. S5 Akoumianaki

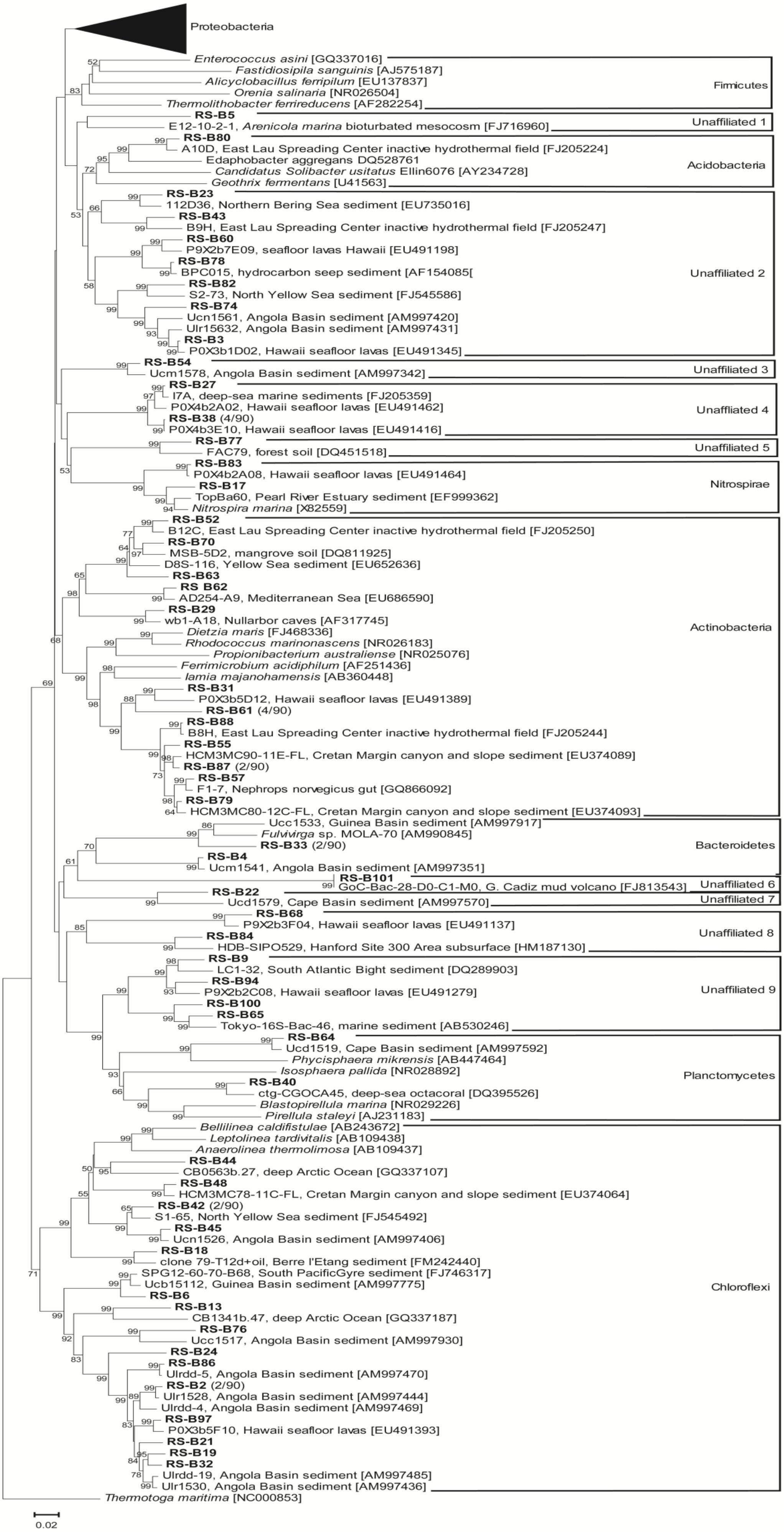

Fig. S6 Akoumianaki

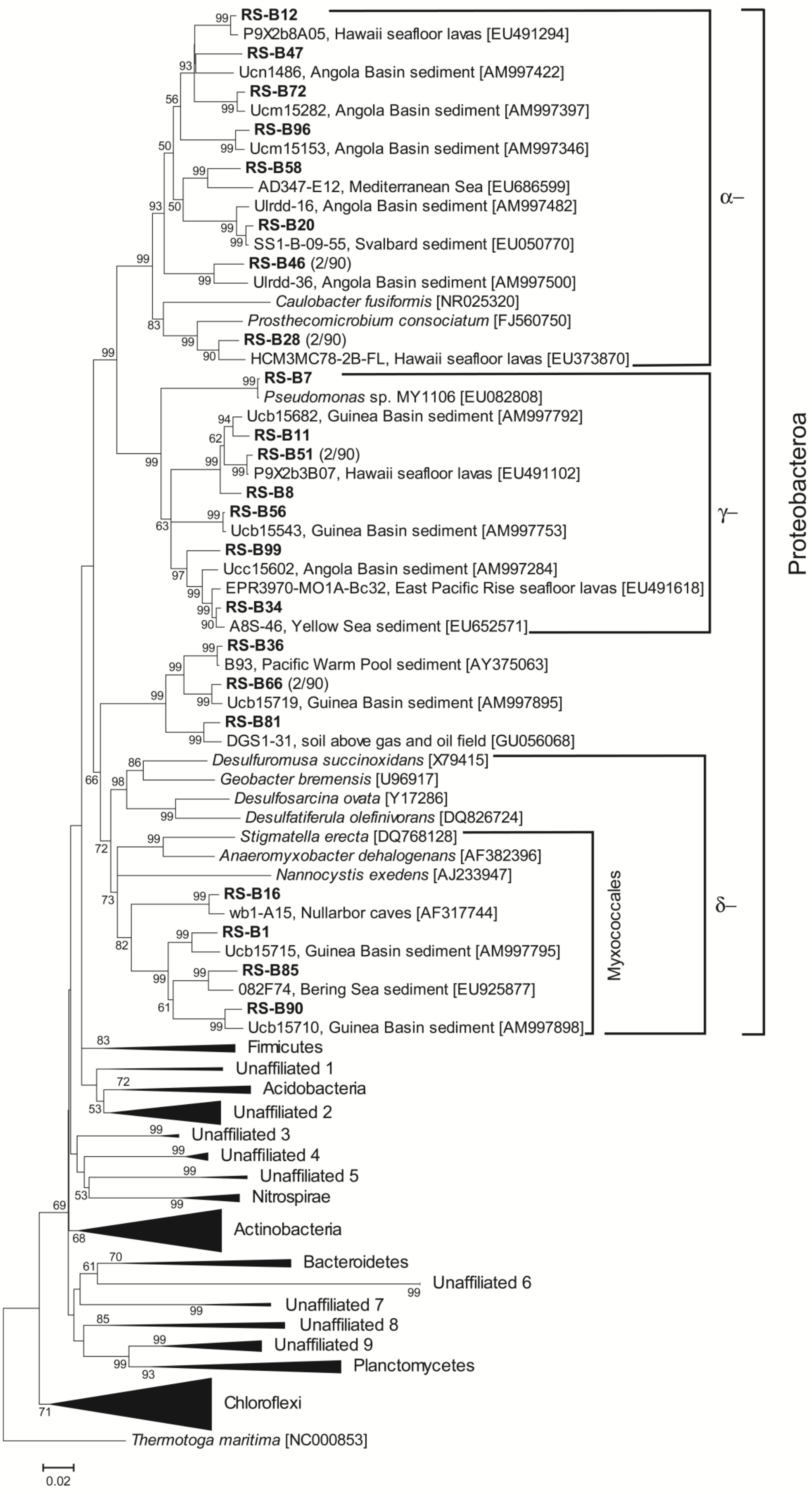

Fig. S7 Akoumianaki
